# Diabetes is associated with elevated mortality, stroke, and osteoradionecrosis in squamous cell carcinomas of the head and neck: a systematic review and meta-analysis

**DOI:** 10.1186/s12903-026-08366-8

**Published:** 2026-04-22

**Authors:** Bulcsú Bencze, Bianca Golzio Navarro Cavalcante, Virág Róna, Bence Szabó, Alexander Schulze Wenning, Péter Hermann, Gábor Varga, Orsolya Németh, Péter Hegyi, Dániel Végh

**Affiliations:** 1 Centre for Translational Medicine, Semmelweis University, Baross u. 22, Budapest, 1085 Hungary; 2 Department of Prosthodontics, Semmelweis University, Szentkirályi u. 47, Budapest, 1088 Hungary; 3 Department of Oral Biology, Semmelweis University, Nagyvárad tér 4, Budapest, 1089 Hungary; 4 Department of Public Dental Health, Semmelweis University, Szentkirályi u. 40, Budapest, 1088 Hungary; 5 Institute for Translational Medicine, Szentágothai Research Centre, Medical School, University of Pécs, Szigeti út 12, Pécs, 7624 Hungary; 6 Division of Pancreatic Diseases, Heart and Vascular Center, Semmelweis University, Üllői út 26, Budapest, 1085 Hungary; 7 Diabetes Dental Working Group, Semmelweis University, Szentkirályi u. 47, Budapest, 1088 Hungary

**Keywords:** Head and neck neoplasms, Survival analysis, Metformin, Glycemic control

## Abstract

**Background:**

Diabetes mellitus has been associated with an increased incidence and potentially with a worse prognosis of squamous cell carcinomas due to chronic inflammation, oxidative stress, and metabolic reprogramming, which promote tumor growth and therapy resistance. Furthermore, it impairs immune response and posttreatment healing, potentially leading to higher complication rates.

**Methods:**

The study protocol was preregistered (CRD42025634653). An electronic search was conducted in MEDLINE, Embase, and Cochrane. Studies on patients with squamous cell carcinomas were included to compare survival metrics and complication rates with and without diabetes. Hazard ratios, odds ratios, and mean differences were extracted. A random-effects model was used, with subgroups based on cancer location.

**Results:**

63 predominantly retrospective studies met the inclusion criteria, with a population of 138,000 individuals. The meta-analysis found statistically significant differences in disease-specific (HR = 1.47 [1.11–1.96]; I^2^ = 60%), overall (HR = 1.31 [1.23–1.39]; I^2^ = 4%), and disease-free survival (HR = 1.58 [1.12–2.24]; I^2^ = 58%); the subgroup analysis revealed that diabetes significantly affected oral and oropharyngeal subsites. Posttreatment stroke (HR = 2.11 [1.15–3.87]; I^2^ = 70%) and osteoradionecrosis rates (HR = 2.2 [1.38–3.53]; I^2^ = 11%) were significantly elevated.

**Conclusion:**

Diabetes is associated with worse disease-specific survival in oral and oropharyngeal subtypes and a twofold higher risk of osteoradionecrosis development in patients with squamous cell carcinoma. Weaker evidence suggests that overall survival and stroke development rate are also affected by diabetes mellitus.

**Supplementary Information:**

The online version contains supplementary material available at 10.1186/s12903-026-08366-8.

## Introduction

Diabetes mellitus (DM) is a group of chronic metabolic disorders characterized by hyperglycemia caused by impaired insulin production or action, leading to the underutilization of glucose as an energy source and overproduction due to inappropriate gluconeogenesis and glycogenolysis [1]. Clinical diagnosis of DM may be based on venous plasma samples with elevated glucose concentrations or increased HbA1c levels in blood samples. There are several clinical categories of DM, such as type 1 and type 2, gestational, and other specific types caused by numerous factors, namely, medication and pancreatic-related issues [2]. Worldwide, 540 million patients live with DM, which is projected to increase to 780 million by 2045 [3].

Head and neck squamous cell carcinomas (HNSCCs) are a significant public health concern, with approximately 650,000 new cases and 330,000 deaths each year, making it the sixth most common cancer worldwide [4]. The annual incidence is projected to increase by 30% by 2030, especially in low Human Development Index (HDI) regions, where risk factors such as excessive alcohol consumption, smoking (or betel chewing), and viral infections (HPV, especially HPV-16) are more prevalent [5–8]. Therefore, a deeper understanding of the factors in cancer progression and outcomes is of paramount importance.

Several studies suggest that patients with DM have an increased risk of developing SSCs compared to non-DM individuals, highlighting the complex interplay between metabolic dysfunction and cancer prognosis [9]. DM elevates systemic cytokine levels, activating metabolic pathways that promote cancer cell growth while impairing the immune response. Subsequently, oxidative stress associated with DM triggers lipid peroxidation and produces reactive oxygen species (ROS), which further contribute to cell damage and promote tumorigenesis [10]. Hyperinsulinemia further promotes tumor progression by increasing Insulin-Like Growth Factor-1, erythroblastic leukemia viral oncogene homolog 2, and 3 receptor expression, thereby promoting cancer cell proliferation and contributing to disease progression [11]. DM also promotes a metabolic shift in cancer cells, increasing their glucose uptake and favoring tumor growth and therapy resistance [12]. Beyond survival, DM compromises surgical site healing and posttreatment recovery, contributing to higher complication rates after cancer treatment, showing DM as a critical factor that should be further investigated [13].

Despite these findings, no comprehensive systematic review or meta-analysis has investigated the impact of DM on survival and posttreatment complication rates in different types of head and neck SSCs. Based on the existing literature, we hypothesize that DM is associated with significantly worse survival outcomes and increased complications in patients with HNSCC.

## Materials and methods

This systematic review followed the Preferred Reporting Items for Systematic Review and Meta-Analysis (PRISMA) 2020 guidelines (Figure S1) [14]. The study protocol was registered on PROSPERO (CRD42025634653).

### Eligibility criteria

Studies were included according to the PECOS framework:

(P) Population: patients diagnosed with SSCs of the head and neck region. (E) Exposure: DM (either type 1 or type 2). (C) Comparison: absence of DM. (O) Outcome measures: survival endpoints, surgical site infection, flap complications, cardiovascular complications, osteoradionecrosis, and length of hospital stay.

(S) Study design: retrospective and prospective observational studies.

Studies on patients with other chronic systemic diseases (e.g., HIV, kidney failure), or another type of cancer, recurrent or previously treated cancer, or distant metastasis at the time of diagnosis were excluded.

### Data sources and searches

The systematic search was conducted across Embase, MEDLINE, and the Cochrane Library in August 2025, without language restrictions or filters, using predefined search terms. EndNote X20.2 (v.7) [15] software and Rayyan.ai [16] were used to manage records and conduct the selection process. To identify all relevant articles, we retrospectively searched the reference lists of included studies and of studies that cited eligible records.

### Study selection

Two review authors (BB & VR) independently screened all identified studies at the title and abstract selection stage, after duplicates were removed, and then at the full-text selection stage. Disagreements in the selection process were resolved by consensus after discussion or consultation with a third reviewer (DV). Reasons for excluding studies during full-text evaluation were recorded and reported. Inter-reviewer agreement (κ) was calculated. All included studies underwent data extraction and risk-of-bias assessment.

### Data extraction

Two reviewers (BB & VR) independently extracted relevant data in duplicate using a standardized electronic form. Data were collected on authors, publication date, country, study period, cohort size, sex ratio, age, disease location, follow-up duration, DM status, HbA1c values, disease definitions, classifications, and staging, as well as predefined clinical outcomes of complications. Disagreements were resolved by consensus after discussion, with a third reviewer (DV) consulted when necessary.

### Risk of bias assessment

The risk of bias assessment was performed in duplicate by two reviewers, using the “Quality in Prognosis Studies” (QUIPS) tool. Study quality was assessed in six domains; the highest risk in any domain determined overall quality. If one domain was high risk or three of six domains were moderate risk, the overall result would be reported as high risk of bias.

### Data synthesis and analysis

Assuming considerable between-study heterogeneity, we used a random-effects model. Effect size measures for survival endpoints, osteoradionecrosis, CVD development, and stroke development are hazard ratios (HRs) with 95% confidence intervals (CIs). To calculate the hazard ratio, we extracted or calculated raw data from studies where available. If only HR without raw data were available, the HR and its 95% confidence interval were utilized (assuming a Wald-type interval). The Paule-Mandel method was used for raw data HR calculation, and the restricted maximum-likelihood estimator was used for direct HR calculation. The Q profile method was used to calculate confidence intervals.

For the length of stay (LOS) outcome, the difference in means (MD) was used as the effect size measure with 95% CI; for flap complications and surgical site infection outcomes, odds ratios (OR) were calculated with 95% CI.

In some cases, multiple data points from the same article were reported, so a multilevel meta-analytic approach was used. We hypothesized that the effects within the studies would be more similar to one another than those in other studies. We also used a three-level model because multiple articles reported data from the same center or hospital. Therefore, data centers were set as random variables [17].

Results were considered statistically significant if the pooled CI did not include the null value. We summarized the meta-analysis findings in forest plots. When the study sample was sufficiently large and not too heterogeneous, the prediction intervals for the results were reported.

Additionally, between-study heterogeneity was assessed using the I2 statistic [18]. Small-study publication bias was assessed by visual inspection of Funnel Plots and by calculating the p-value of the classic Egger’s test for MD effect size [19]. We assumed a possible small study bias if the *p*-value was less than 10%.

Subgroup analyses were performed using information on disease location [20].

All statistical analyses were performed with R (version 4.3.0) [21] using the meta (version 6.2.1) [22] package for basic meta-analysis calculations and plots, and the metafor (versions 3.8.1 & 4.2.0) [23] package for multilevel models and meta-regression.

### Certainty of evidence

Certainty of evidence was assessed by two reviewers (BB and VR) for all outcomes using the Grading of Recommendations Assessment, Development, and Evaluation (GRADE) tool.

## Results

### Search and selection

**Fig. 1:**
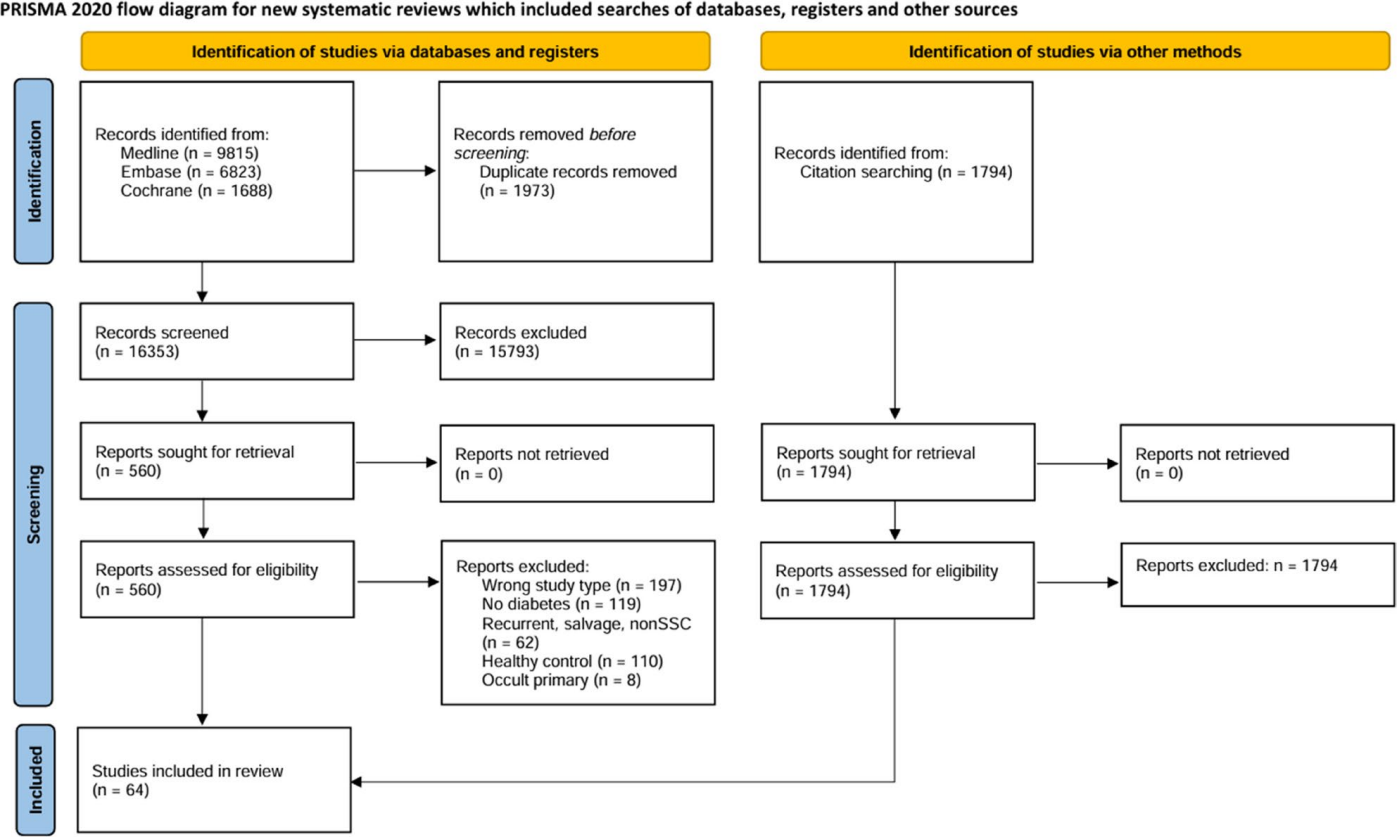
PRISMA flow chart presenting the course of study selection procedure

Altogether, 18,326 records were identified, of which 16,353 remained after duplicate removal. Title and abstract selection was conducted (κ = 0.82), resulting in 560 studies selected for full-text review (κ = 1). After the citation search procedure, 63 articles were included in the final pool. (Fig. 1) [23].

### Characteristics of included studies

The 63 articles contained 138,000 individuals, including 22,800 patients with DM. They were published between 2006 and 2024, with populations from various countries, including Taiwan [24–42], the United States [43–51], China [52–65], Canada [66], Hungary [67, 68], Germany [69, 70], Pakistan [71], Israel [72], Republic of Korea [73–75], the United Kingdom [76], Spain [77], Denmark [78], Austria [79], Serbia [80], Italy [81], Japan [82–84] and Australia [85, 86] (Table 1). Four studies were prospective, while 59 were retrospective. Age ranged from 18 to 92 years (mean 58.7), and the population was predominantly male (72.7%). Altogether, 19 studies investigated patients with type 2 DM; 2 included both type 1 and type 2 DM, and 42 did not mention the type of DM. HbA1c levels in blood samples were used to assess glycemic control in only 6 studies. The studies either included SSCs of the whole head and neck region (HNSSC) or SSCs of specific locations, such as the oral, oropharyngeal, hypopharyngeal, laryngeal, and nasopharyngeal regions.

**Table 1:** Basic characteristics of included studies

Author (year)	Country	Number of patients (female %)	Age in years (mean ± SD or (range))	Location	Type of DM	Means of DM diagnosis	Means of cancer diagnosis	Stages in % (1/2/3/4)	Basis of staging	Treatment (OP/OP+CCRT/CCRT)
Chun-Yuan Chao [24]	Taiwan	3600 (5.8%)	NR	OSCC	Mixed	Blood sample HbA1c	NR	19.3/20.7/13.1/46.5	Ajcc 7th	50.4/47.5/5.1
Ze Yun Tay [25]	Taiwan	358 (8%)	53.3 ± 10.3	OSCC	NR	Blood sample HbA1c	Histological confirmation	NR	Ajcc 7th	45.57/54.43
Han-Chieh Cheng [26]	Taiwan	191 (7.3%)	50.9 ± 10.4	OSCC	NR	Blood sample HbA1c	Diagnostic codes	0/0/0/100	Ajcc 7th	NR
Andrew Foreman [66]	Canada	2498 (25%)	NR	OSCC, OPSCC, HPSSC, LaSCC	type 2 DM	NR	NR	Stage 1&2: 36 Stage 3&4: 64	Ajcc 7th	20/15/65
Krupal B. Patel [43]	USA	274 (29.2%)	60.3 ± 12.4	OSCC	NR	NR	NR	22.3/24.2/19.4/34.1	Ajcc 7th	52.8/47.2/0
Márta Ujpál [67]	Hungary	100 (48%)	56.4 (38–77)	OSSC	type 2 DM	Fasting blood glucose	Histological confirmation	NR	T/N/M	0/100/0
Ching-Nung Wu [27]	Taiwan	5673 (8.4%)	NR	OSSC	NR	NR	NR	23.3/25.3/13.8/37.6	Ajcc 7th	54.8/45.2/0
Cheng-Hsien Wu [28]	Taiwan	372 (11.5%)	55 ± 13.5	OSSC	NR	Fasting blood glucose	Histological confirmation	NR	Ajcc 7th	NR
Xin Hu [52]	China	407 (14.7%)	53.7 ± 9.1	OSSC	type 2 DM	Medical records	Histological confirmation	NR	Ajcc 7th	NR
Christoph Klingelhoffer [69]	Germany	400 (33.8%)	62.3 ± 11.2	OSSC	NR	NR	NR	44/27.1/6/22.9	Uicc 7th	100/0/0
Linda X. Yin [44]	USA	406 (10%)	NR	OPSSC	NR	NR	NR	79/17/4/0	Ajcc 8th	24/76/0
K. Zaoui [70]	Germany	251 (22.7%); 173 (11%)	59.7 ± 9.8; 61.8 ± 10.2	OSSC, LaSCC, OPSCC	Majority type 2 DM	Medical records, blood sample HbA1c, fasting glucose	NR	7.9/10.4/15.1/66.9; 17.9/17.9/17.3/46.8	Uicc	NR
Yumna Adnan [71]	Pakistan	386 (23.3%)	50.19 ± 13.36	OSSC	Majority type 2 DM	Medical records	Histological confirmation	NR	NR	NR
Hui Liu [53]	China	934 (16.3%)	NR	NPSSC	type 2 DM	Fasting blood glucose	NR	NR	Chinese Fuzhou	0/0/100
Pu-Yun OuYang [54]	China	4294 (24%)	NR	NPSSC	NR	Fasting blood glucose	Histological confirmation	NR	Ajcc 7th	0/0/100
Hao Peng [55]	China	1489 (23.5%)	NR	NPSSC	NR	Fasting blood glucose	Histological confirmation	NR	Ajcc 7th	0/0/100
Xing-Si Peng [56]	China	558 (23.65%)	NR	NPSSC	type 2 DM	Fasting blood glucose	Histological confirmation	NR	Uicc (2002)	0/0/100
Vlad C. Sandulache [45]	USA	205 (14%)	63	LaSSC	NR	NR	Histological confirmation	NR	Ajcc	NR
Yuval Nachalon [72]	Israel	265 (18%)	64 ± 11.8	LaSSC	NR	NR	NR	35/24/19/22	NR	7/4/89
Yung-An Tsou [57]	China	141 (NR)	51.7	HPSSC	NR	NR	NR	NR	T/N/M	0/0/100
Ming-Hsien Tsai [29]	Taiwan	233 (5.6%)	NR	HNSSC	type 2 DM	NR	NR	NR	Ajcc 8th	79/21/0
Minsu Kwon [73]	Republic of Korea	1151 (13.9%)	NR	HNSSC	type 2 DM	Blood sample HbA1c	Histological confirmation	NR	NR	NR
Pei-Ling Tang [30]	Taiwan	1660 (8.37%)	NR	HNSSC	NR	Medical records	Diagnostic codes	NR		54.5/0/65.5
Hsuan-Chih Kuo [31]	Taiwan	556 (6.5%)	64	HNSSC	NR	NR	NR	NR	Ajcc	0/24/76
Aastha Sobti [76]	United Kingdom	151 (26%)	61 ± 12	HNSSC	type 2 DM	Medical records	Histological confirmation	NR	NR	NR
Yung-An Tsou [32]	Taiwan	141 (NR)	64.5	HPSSC	NR	Medical records	NR	NR	T/N/M	NR
Delong Li [58]	China	399 (37.3%)	NR	OSSC	NR	NR	Histological confirmation	NR	Ajcc 8th	NR
Young-Hoon Joo [74]	Republic of Korea	229 (11.6%)	56.8 (20–78)	HNSSC	NR	Medical records	Histological confirmation	NR	Ajcc 2002	NR
Sophia A. Ederaine [46]	USA	190 (16.8%)	70.5 ± 10.24	HNSSC	Mixed	Blood sample HbA1c	NR	16.2/8.9/14.5/60.3	NR	84.7/NR/NR
Daniel E. Spratt [47]	USA	1745 (12.9%)	56 (25–91)	OPSSC	NR	Medical records	Histological confirmation	NR	Ajcc	NR
Francesc Xavier Aviles-Jurado [77]	Spain	71 (8.5%)	NR	HNSSC	type 2 DM	Blood sample HbA1c, fasting glucose	Histological confirmation	Stage 1&2: 33.8 Stage 3&4: 66.2	NR	50.7/0/49.3
Milap D. Raikundalia [48]	USA	31,075 (NR)	63.4 ± 11.85	HNSSC	NR	Medical records	Diagnostic codes	NR	NR	NR
Xinglong Chen [59]	China	88 (18.8%)	62.5	LaSSC	NR	NR	Histological confirmation	NR	NR	NR
Christina Eder-Czembirek [79]	Austria	85 (30.6%)	58.4 (25–79)	OSSC	NR	Medical records	Medical records	NR	Uicc 7th	0/100/0
Shih-An Liu [33]	Taiwan	994 (6.8%)	51.5 ± 11.87	OSSC	NR	NR	NR	22/31.4/16.1/30.5	Ajcc	NR
Chun-Yue Ma [60]	China	376 (51.3%)	73.1 ± 3.4	OSSC	NR	Medical records	Histological confirmation	Stage 1&2: 62 Stage 3&4: 38	NR	NR
Jelena Sotirović [80]	Serbia	277 (11.2%)	59.48 (34–82)	LaSSC	NR	NR	Histological confirmation	NR	NR	NR
Chiara Bianchini [81]	Italy	168 (14.3%)	65.7 ± 11.3	HNSSC	type 2 DM	Medical records	Histological confirmation	6/22/28.6/45.4	NR	NR
Chengwen Gan [61]	China	632 (22%)	57 (24–92)	HNSSC	NR	NR	NR	Stage 1&2: 53.8 Stage 3&4: 46.2	NR	NR
Chao-Hsien Wang [34]	Taiwan	535 (98.7%)	51.7 ± 10	HNSSC	NR	NR	NR	Stage 1&2: 28.5 Stage 3&4: 71.5	Ajcc	NR
Yung-Hsuan Chen [35]	Taiwan	2599 (24.9%)	50 ± 12	NPSSC	NR	NR	Diagnostic codes	Stage 1&2: 31.7 Stage 3&4: 68.9	Ajcc	NR
Qigen Fang [62]	China	856 (31.1%)	NR	OSSC	type 2 DM	Medical records	Medical records	0/32.6/51.9/15.5	Ajcc 8th	NR
Wenlu Li [63]	China	469 (24%)	NR	OSSC	NR	NR	NR	0/25/42.9/32.1	Ajcc 8th	NR
Zhonglong Liu [64]	China	309 (43%)	68.6 ± 5.7	OSSC	type 2 DM	Medical records	NR	Stage 1&2: 16.8 Stage 3: 19.7 Stage 4: 63.4	T/N/M	0/100/0
Shih-Lun Lo [36]	Taiwan	158 (8.2%)	52.4 ± 10.11	HNSSC	NR	NR	NR	11.4/19/10.8/58.2	NR	70.4/29.6
Tu, Cheng Hung [37]	Taiwan	623 (3%)	NR	HNSSC	NR	NR	NR	NR	NR	NR
Pi-Chieh Lin [38]	Taiwan	584 (7.8%)	55 ± (48–61)	HNSSC	NR	NR	NR	NR	NR	49.7/50.3/0
Ran Ito [39]	Taiwan	345 (5.2%)	50.5 ± 10.4	OSSC	NR	NR	Histological confirmation	11.3/34.5/25.8/28.4	Ajcc 2010	51.6/48.4/0
Hikaru Kubota [82]	Japan	616 (17%)	NR	HNSSC	NR	NR	Histological confirmation	Stage 1&2: 60 Stage 3&4: 40	T/N/M	0/100/0
Hans P. Sathasivam [85]	Australia	325 (22.8%)	64.2 ± 10.8	HNSSC	type 2 DM	Medical records	Histological confirmation	13.5/34.8/29.8/21.8	T/N/M	12.3/60.4/27.3
Feng-Che Kuan [40]	Taiwan	5172 (10.2%)	55.28 ± 11.02	OSSC	NR	Medical records	Diagnostic codes	NR	NR	NR
Chia-Fan Chang [41]	Taiwan	5618 (6.7%)	NR	OSSC	NR	Medical records	Diagnostic codes	NR	NR	NR
Min-Chi Chen [42]	Taiwan	3016 (28.2%)	52.52 ± 11.41	NPSSC	NR	Medical records	Diagnostic c,,,odes	NR	NR	NR
Amrita Mukherjee [49]	USA	1829 (25.37%)	NR	HNSSC	NR	Medical records	Histological confirmation	Stage 1&2: 35.21 Stage 3&4: 49.54	T/N/M	39.64/18.97/26.63
Daniel Addison [50]	USA	326 (25.1%)	58.8 ± 11.9	HNSSC	NR	NR	Histological confirmation	NR	NR	NR/NR/92.5
Lova Sun [51]	USA	35857 (1.1%	NR	HNSSC	type 2 DM	Medical records	Diagnostic codes	25.2/27.8/17.7/19.6	T/N/M	NR
Eun Joo Kang [75]	Republic of Korea	7467 (19.7%); 7730 (19.7%)	NR	HNSSC	NR	Medical records	Diagnostic codes	NR	NR	NR
Chiyoko Makita [83]	Japan	111 (2.7%)	NR	LaSSC, HPSSC	NR	Fasting blood glucose	Histological confirmation	43.2/42.3/9/3.8	T/N/M	NR
Yaqing Mao [65]	China	154 (44.2%)	NR	OSSC	type 2 DM	Blood sample HbA1c, fasting glucose	Histological confirmation	13/16.9/41.6/27.3	Ajcc 8th	NR
Rhiannon Mellor [86]	Australia	235 (10.8%)	59 ± 7.5	HNSSC, NPSSC	NR	Medical records	Histological confirmation	Stage 2&3: 39.1 Stage 4: 53.4	NR	NR
Lars Merring-Mikkelsena [78]	Denmark	250 (22.8%)	NR	HNSSC	NR	Medical records	Histological confirmation	NR	Ajcc 7th	NR
Shin Midorikawa [84]	Japan	70 (17.9%)	64.5 ± 12.8	NPSSC	type 2 DM	Medical records	Histological confirmation	4.3/19.1/40.4/36.2	Ajcc 8th	NR
Gábor Dénes Répássy [68]	Hungary	293 (15.7%)	64.05 ± 7.28	LaSSC	type 2 DM	Medical records	Histological confirmation	NR	Ajcc	NR

*Abbreviations*: *NR* Not reported, *SSC* Squamous cell carcinoma, *La* Laryngeal, *HP* Hypopharyngeal, *HN* Head and neck, *NP* Nasopharyngeal, *O* Oral, *OP* Oropharyngeal

### Risk of bias

Only one study was classified as having a high overall risk of bias, due to moderate risks with three domains [32]. In the included studies, concerns arose regarding the definition and measurement of the prognostic factor, as many did not specify the type of DM or the method of diagnosis. Additionally, the description and adjustment for confounding factors were limited (Sup. Figure 1).

### Results of individual studies and synthesis of results

#### Disease-specific survival

**Fig. 2:**
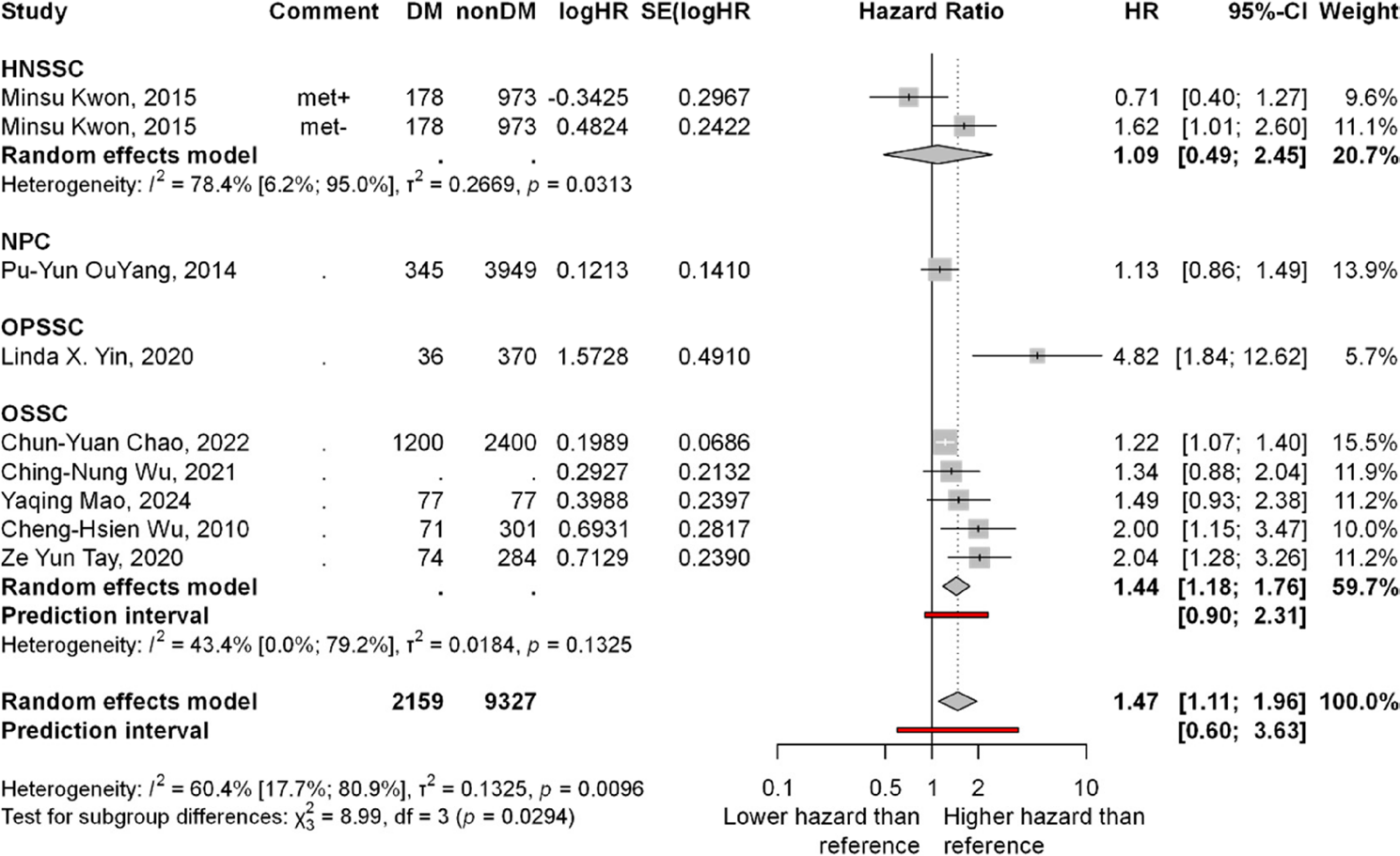
Forest plot showing disease-specific survival in patients with diabetes compared with those without, stratified by carcinoma location. Abbreviations: SSC: Squamous cell carcinoma; HN: Head and neck; NP: Nasopharyngeal; O: Oral; OP: Oropharyngeal

Ten studies reported disease-specific survival data across several subtypes. The highest and lowest univariate survival rates were 4.82 [1.84–12.61] in oropharyngeal SSC patients and 0.71 [0.4–1.29] in HNSSC patients. Four studies found statistically significant differences in survival between the groups, and all found worse survival outcomes in patients with DM. Eight articles were included in the quantitative analysis and showed statistically significant differences between patients with and without DM, with an overall HR of 1.47 [1.11–1.96] and substantial heterogeneity (I2 = 60% [17.7%-80.9%]) (Fig. 2). The analysis found a statistically significant difference in the oral SSC group (HR = 1.44 [1.18–1.76]; I^2^ = 43.4% [0%-79%]). One study differentiated between patients on metformin; patients on the medication had higher survival rates (HR = 0.71 [0.40–1.27]) compared to patients not taking the drug (HR = 1.62 [1.01–2.6]). Datasets of multivariate survival metrics are available in the supplementary material (Sup. Table 1).

#### Overall survival

**Fig. 3:**
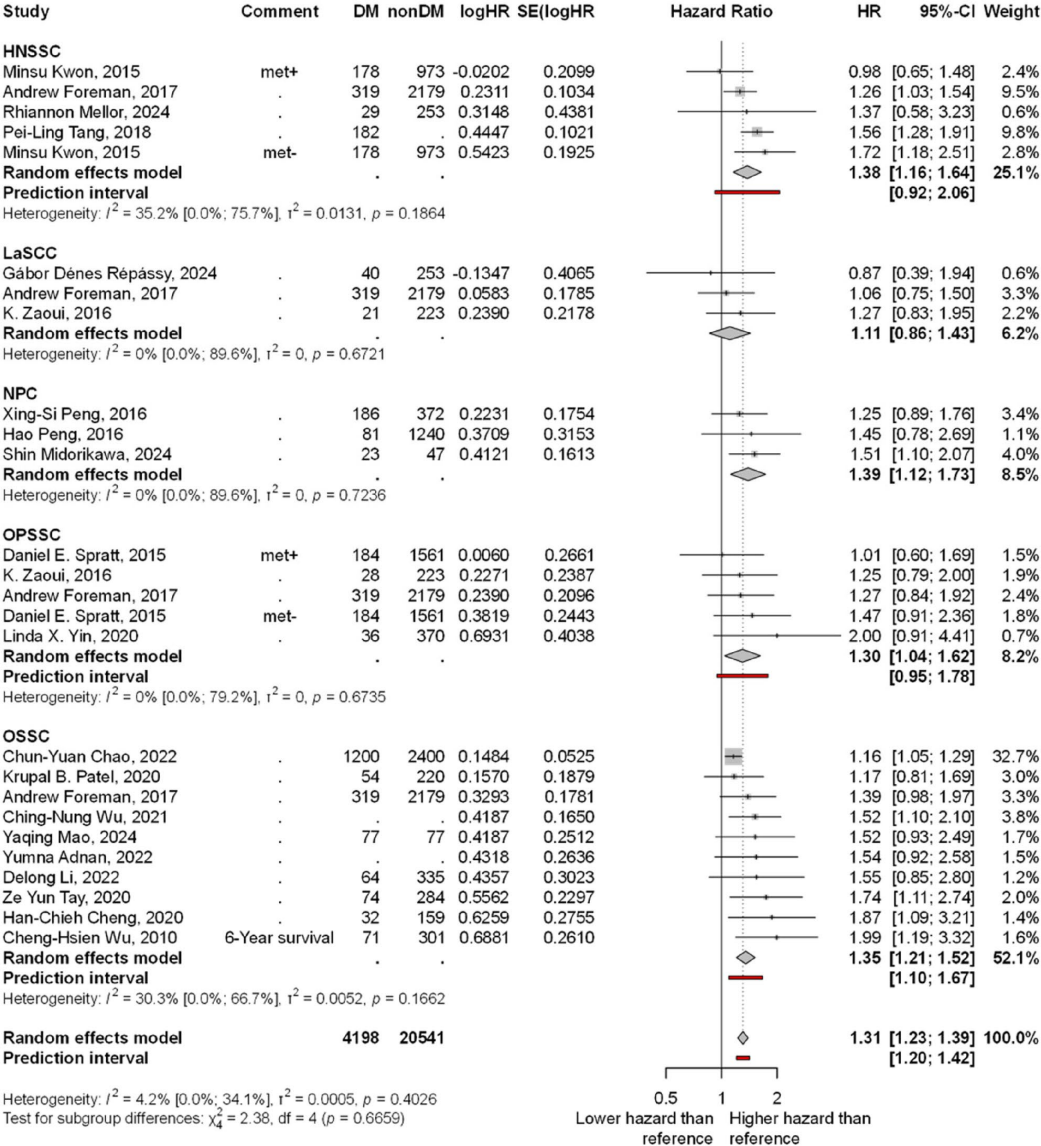
Forest plot showing overall survival in patients with diabetes compared with those without, stratified by carcinoma location. Abbreviations: SSC: Squamous cell carcinoma; La: Laryngeal; HP: Hypopharyngeal; HN: Head and neck; NP: Nasopharyngeal; O: Oral; OP: Oropharyngeal

Thirty-one studies reported data on overall survival across all cancer groups. The highest and lowest HR values reported were HR = 2.57 [1.09–6.07] and HR = 0.95 [0.59–1.5]. Eight univariate comparisons showed statistically significant differences, all favoring worse survival outcomes in the DM group. For the meta-analysis, we used 15 articles and found statistically significant differences between the groups, with an HR of 1.31 [1.23–1.39] and low heterogeneity (I2 = 4.2% [0%-34%]) (Fig. 3). Subgroup analysis by location showed statistically significant differences in the oral, oropharyngeal, and HNSSC groups, with HRs of 1.35 [1.21–1.52], 1.30 [1.04–1.62], and 1.38 [1.16–1.64], respectively. Two studies compared the impact of metformin on overall survival, with those taking metformin in the HNSSC and oropharyngeal groups presenting better survival outcomes with values of HR = 0.98 [0.65–1.48] and HR = 1.01 [0.60–1.69] than those not taking metformin with values of HR = 1.72 [1.18–2.51] and HR = 1.47 [0.91–2.36], respectively. Multivariate analysis found lower survival chances in patients with DM in all but one study (Sup. Table 2).

#### Disease-free survival

Fourteen articles reported data on disease-free survival, with the highest and lowest HR values being 2.96 [1.88–4.68] and 1.1 [0.43–3.05], respectively. The overall results of the meta-analysis showed a statistically significant difference (HR = 1.58 [1.12–2.24]) with substantial heterogeneity (I2 = 58% [0%-84%]) (Sup. Figure 2). Multivariate analysis results were similar to the overall survival outcome (Sup. Table 3).

#### Distant metastasis-free survival

Six articles reported data on distant metastasis-free survival. The highest and lowest HR values recorded were HR = 3.05 [1.23–7.37] and HR = 1.19 [0.78–1.89], respectively. The overall meta-analysis did not show a statistically significant result, with an HR of 1.28 [0.96–1.70] (Sup. Figure 3). One article investigated the effect of metformin on distant metastasis-free survival and found significant differences between the two groups. Patients on metformin had a significantly lower risk of developing distant metastases, with an HR of 0.93 [0.46–1.90], compared to an HR of 1.78 [1.01–3.13]. All studies reporting multivariate analysis results found lower survival rates among patients with DM (Sup. Table 4).

#### LOS, flap complication, surgical site infection, stroke, CVD, and osteoradionecrosis development

Three studies reported on the short-term posttreatment outcome of LOS. The meta-analysis found no statistically significant differences between the groups with a value of 3.29 [− 0.08–6.65] days (Sup. Figure 4).

Six articles reported flap complication rates; three found statistically significant differences between groups. The meta-analysis showed no statistically significant differences between the groups, with an overall value of OR = 1.37 [0.74–2.54]. Subgroup analysis by location was possible for the HNSSC group, with an OR of 1.54 [0.31–7.73] (Sup. Figure 5). One article reported multivariate analysis results (OR = 2.885 [1.497–5.56]).

Twelve articles investigated the impact of DM on surgical site infection rates. The meta-analysis of univariate data found no statistically significant differences, with an overall outcome of OR = 1.63 [0.98–2.69] (Sup. Figure 6). Subgroup analysis was possible for the HNSSC group (OR = 1.62 [0.86–3.04]). Seven articles reported multivariate results, and all found statistically significant differences (Sup. Table 5).

**Fig. 4:**
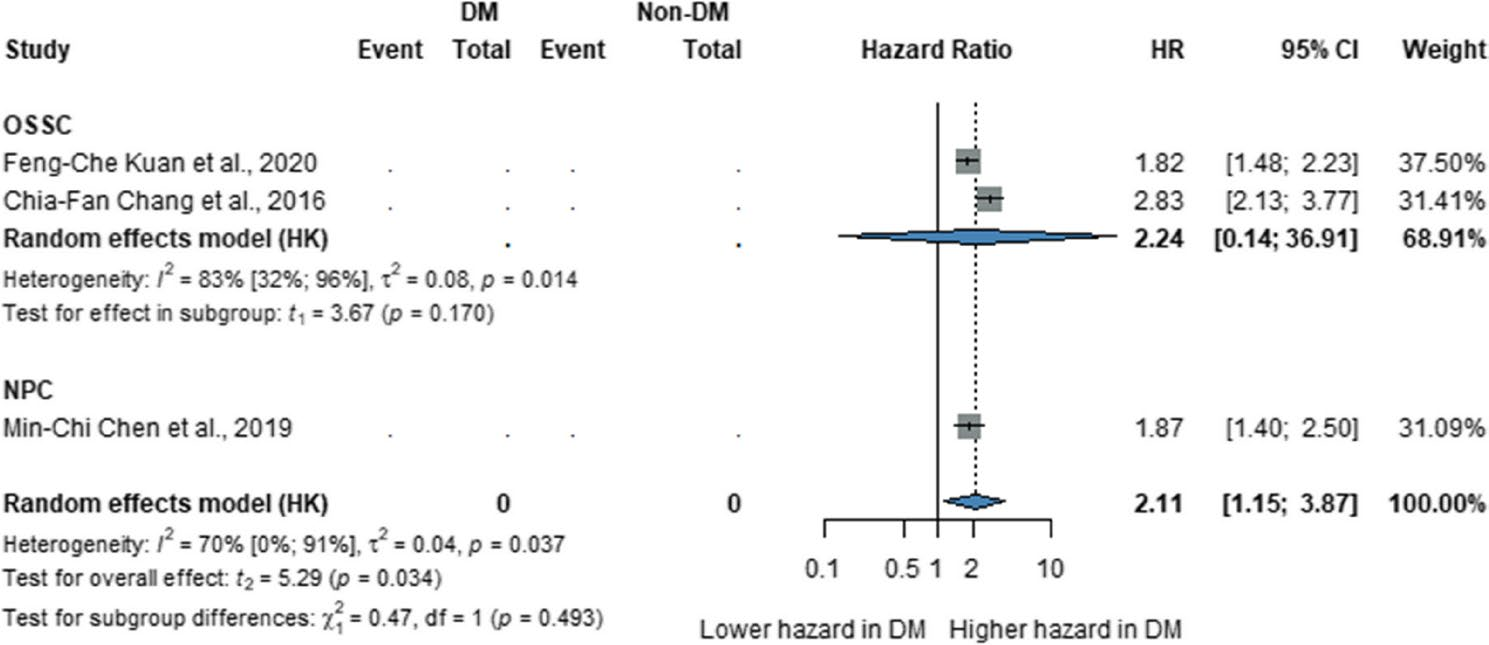
Forest plot presenting stroke development in patients with diabetes compared to patients without diabetes, with subgroups based on different carcinoma locations. Abbreviations: SSC: Squamous cell carcinoma; NP: Nasopharyngeal; O: Oral

Seven articles reported on posttreatment stroke development outcomes. The meta-analysis showed a statistically significant difference between the groups, with an overall HR of 2.11 [1.15–3.87] (Fig. 4). Five articles presented multivariate analysis results, all five found statistically significant differences (Sup. Table 6).

Four articles investigated the impact of DM on posttreatment CVD complications, and one article reported univariate analysis outcomes that also differentiated between patients taking and not taking antidiabetic medication. Patients taking medication had a lower risk of developing CVD complications (HR = 1.93 [1.21–2.62]) compared with patients not taking medication (HR = 3.52 [1.32–6.77]). Three articles reported multivariate analysis outcomes, two of which presented statistically significant differences between the groups (Sup. Table 7).

**Fig. 5:**
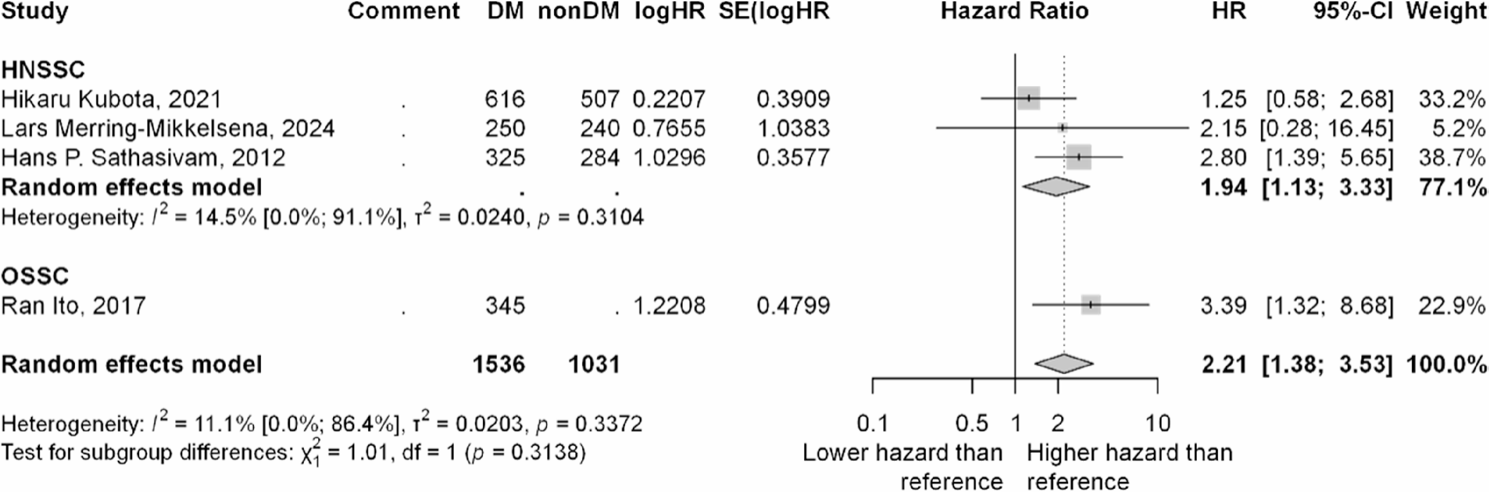
Forest plot comparing osteoradionecrosis in patients with diabetes to those without, with subgroups by carcinoma location. Abbreviations: SSC: Squamous cell carcinoma; HN: Head and neck; O: Oral

Four studies reported data on osteoradionecrosis outcome. The meta-analysis showed a statistically significant difference between the groups, with an overall HR of 2.2 [1.38–3.53] (Fig. 5), and two articles reported multivariate analysis results, showing statistically significant differences (Sup. Table 8).

#### Inconsistency and publication bias

Publication bias was analyzed for the outcome overall survival due to the required number of studies, where significant publication bias may be present (*p*-value = 0.0365; Sup. Figure 7).

#### Certainty of evidence

The certainty of evidence for disease-specific, distant metastasis-free survival and osteoradionecrosis was moderate. Overall survival, disease-free survival, flap complication rate, LOS, and surgical site infection rate were low, and the rate of stroke development had very low certainty of evidence (Sup. Figure 8).

## Discussion

We performed a systematic review and meta-analysis of 63 observational studies involving approximately 138,000 patients to investigate the impact of DM on survival and posttreatment complication rates in patients with SSCs of the head and neck. Although several studies reported multivariate analyses, the included covariates differed considerably between studies, most commonly including age, tumor stage, smoking status, alcohol consumption, comorbidity indices, and treatment modality. Due to this heterogeneity, only univariate effect estimates were pooled quantitatively (Sup. Table 9). The meta-analysis demonstrated that patients with DM experienced significantly poorer disease-specific, overall, and disease-free survival, corresponding to 1.5-, 1.3-, and 1.6-fold reductions in survival probability, respectively. In contrast, no statistically significant difference in distant metastasis–free survival was observed between patients with and without DM. Regarding short-term complications, statistically significant differences were observed only in the incidence of stroke and osteoradionecrosis among patients with DM, with relative risks increased by 2.1-fold and 2.2-fold, respectively. Although not statistically significant, the incidence of surgical site infections was clinically relevant, occurring 1.6 times more frequently in patients with DM.

A previous meta-analysis investigated the impact of DM on the risk of head and neck cancer development and found statistically significant associations between oral SSC development and DM (RR = 1.22 [1.01–1.47]), whereas, in other locations, the differences were not significant, except in the East Asian population (RR = 1.46 [1.21–1.77] [87]. One recent meta-analysis addressed the impact of DM on overall survival in patients with oral and oropharyngeal SSCs and found significant differences in survival (HR = 1.69 [1.29–2.22]) [88]. However, the study did not investigate subtypes separately and combined univariate and multivariate regression analyses, making the results difficult to interpret.

Disease-specific survival is the most difficult outcome to provide direct evidence of the influence of the prognostic factor. Subgroup analysis was only possible in the oral SSC subtype (HR = 1.48 [1.14–1.91]); the remaining studies from other subtypes did not affect the magnitude of the association. One study investigated the direct effect of glycemic control and found significant differences between well-controlled (~ 6.5% HbA1c) and poorly controlled (8.5% HbA1c) individuals, with HRs of 1.51 (1.15–1.99) and 2.22 (1.62–3.01), respectively [24]. Metformin was also investigated as a beneficial medication, with patients on the medication showing better survival than non-DM patients (HR = 0.71 [0.40–1.27]) [73]. Overall survival provides less direct evidence of disease progression, but offers a more complete picture of patient longevity regardless of cause. We found statistically significant differences in overall survival regardless of location (HR = 1.31 [1.22–1.41]), and similar results across specific subtypes, including oral, oropharyngeal, and mixed HNSSC. Although the literature was limited in laryngeal and nasopharyngeal cases, no statistically significant differences were found. Numerous pathophysiological factors explain the smaller impact of DM on survival and the lower risk of development in nasopharyngeal and laryngeal locations, namely, less direct exposure to hyperglycemia. Meanwhile, the localized effects of hyperglycemia are more pronounced in oral and oropharyngeal subtypes, leading to a pro-inflammatory environment with bacterial dysbiosis and periodontitis [89, 90]. Additionally, the association of nasopharyngeal SSCs with EBV suggests that viral oncogenesis plays a more significant role than hyperglycemia [91]. The beneficial effect of metformin can also be observed in overall survival in nasopharyngeal and mixed HNSSC subtypes. A systematic review and meta-analysis by Jiao et al. compared HNSSC patients taking and not taking metformin and found that overall survival was better in patients on the medication (HR = 0.87 [0.76–0.99]) [92]. Preclinical studies show that metformin targets cancer-initiating cells by activating AMPK and inhibiting mTOR, and it increases the activity of natural killer cells by inhibiting the chemokine ligand CXCL1, suggesting an immunomodulatory effect [93, 94]. Evidence shows that hyperglycemia promotes HNSSC progression by supporting tumor cell proliferation, limiting apoptosis, and increasing invasion potential by glucose transporter upregulation, growth factor activation, and stimulation of matrix metalloproteinase; additionally, chronic hyperglycemia promotes inflammation, oxidative stress, and epigenetic modifications, further supporting tumor aggressiveness and therapy resistance [95]. These mechanisms contribute to worse survival outcomes and recurrence in patients with DM, which is in line with the findings of this article, highlighting the need for glycemic control and optimal medication in cancer management.

LOS helps to optimize hospital capacity, manage resources, and reduce hospital-acquired infections. Unfortunately, the impact of DM on LOS in patients with HNSSC is under-researched [96]. Our evidence on the impact of DM on LOS is limited, and we found a statistically nonsignificant difference (~ 3.3 days). However, the difference may be clinically relevant, as the average LOS in HNSSC patients ranges from 3 to 8 days [97]. A critical study investigated the effect of glycemic control on LOS in patients with various head and neck malignancies and found that well-controlled individuals had LOS similar to those of non-DM individuals. In contrast, poorly controlled patients had a significantly longer LOS and total hospital costs [98]. Our analysis of flap complication rates found no statistically significant differences between the groups, regardless of location. A previous meta-analysis on this topic found significant associations between DM and flap complication rates (RR = 1.83 (1.18–2.85)] in patients with all kinds of head and neck malignancies, without restriction on location, recurrent or salvage surgeries; however, the overall results may be influenced by studies with low number of patients and events recorded (e.g., one flap complication recorded in the DM group yielding RR = 28.24) [99]. Surgical site infection is a well-researched short-term complication with a high rate, which is significantly affected by DM [100]. Studies have shown that perioperative glycemic levels are an independent risk factor for surgical site infections, and stringent glycemic control may lower the risk of infection in patients with DM [101]. A recent meta-analysis found a serious association between surgical site infection and DM in patients with various types of head and neck malignancies (OR = 3.00 [2.12–4.16]). However, including recurrent diseases and salvage surgeries increased the risk of infection, yet there is an apparent association between DM and surgical site infection [102]. Our analysis yielded a statistically non-significant result (OR = 1.63 [0.98–2.69]). However, all studies reporting multivariate results found significant associations ranging from 1.4- to 6-fold higher risks of infection. Posttreatment stroke development in HNSSC patients is primarily associated with radiotherapy due to damage to neighboring vasculature, leading to several conditions such as carotid and vertebral artery stenosis [103]. Unfortunately, DM further elevates the risk of stroke development in patients through hyperglycemia-associated systemic inflammation and oxidative stress [104]. Our analysis found that DM doubles the risk of posttreatment stroke, which is in line with the literature. Regarding CVD complications, two of three studies showed statistically significant differences between patients with and without DM. Several studies investigated the beneficial effects of antidiabetic medication on CVD complications, reporting promising results, such as sotaglifozin, which may be a recommended medication for DM patients with CVD risk factors [105]. We have found moderate evidence on the impact of DM on posttreatment osteoradionecrosis, with four studies reporting significant differences, ultimately showing a two times higher risk of development in patients with diabetes. Hence, this finding was based on only four studies; the result should be interpreted cautiously. Patients receiving either bisphosphonates or radiation therapy are at risk of posttreatment osteoradionecrosis, and the presence of DM further compromises bone healing and integrity, underscoring the importance of glycemic control in reducing the risk in this population [106]. Importantly, the included studies demonstrated substantial heterogeneity in the definition and characterization of diabetes mellitus. Only a limited proportion of studies reported HbA1c levels or measures of glycemic control, preventing subgroup analyses based on metabolic control. As poor glycemic control is associated with impaired immune function, increased inflammation, and delayed wound healing, this limitation may contribute to residual confounding and should be considered when interpreting the observed associations. ​Limited research exists on new and popular antidiabetic medications (glucagon-like peptide-1 receptor agonists), such as semaglutide (Ozempic) and liraglutide (Saxenda), and some studies have concluded that they have no significant impact on overall survival [107]. On the other hand, SGLT-2 inhibitors were associated with a 54% reduced risk of all-cause mortality (RR 0.46) and a significant reduction in heart failure hospitalization in patients with other types of cancer [108].

### Strengths and limitations

The main novelty of this systematic review and meta-analysis is the separation of SSC subtypes in the head and neck region, the inclusion of multiple survival composite outcomes, the distinction between univariate and multivariate outcomes, and the investigation of post-treatment complication rates affected by DM. We adhered to our preregistered protocol for objectivity and rigorous methodology. Excluding recurrent diseases, cancers with pre-existing distant metastases, salvage surgeries, and other cancers in the head and neck region increases the strength, evidence, and generalizability of the results compared to previous meta-analyses. In terms of limitations, several studies lacked a definition of the prognostic factor and the diagnostic criteria. Little information was available on glycemic control quality, making the impact of well-controlled glycemic levels difficult to interpret. Different adjustments were applied in multivariate results, limiting the number of studies that could be pooled. Several modifying factors were investigated, including different disease stages and population characteristics, as well as surgical and chemotherapy/radiotherapy protocols, which resulted in greater heterogeneity.

### Implications for clinical practice

It is essential to translate research into general practice [104]; therefore, regular screening of patients with hyperglycemia to identify any potentially cancerous or precancerous lesions is strongly recommended. DM appears to be an adverse prognostic and complication-associated comorbidity, and careful metabolic assessment and multidisciplinary management may be warranted. Therefore, patients may benefit from systemic glycemic measurements, which would help identify underlying or poorly controlled DM and provide the opportunity for further metabolic management when necessary.

### Implications for research

We suggest that prospective studies investigate the effects of different glycemic control qualities, with rigorous HbA1c measurements. Furthermore, evidence on the impact of metformin, and especially on GLP-1 receptor agonists and SGLT-2 inhibitors, is lacking. More prospective data collection is needed to increase the certainty of the evidence, including frequent HbA1c measurements during follow-up, strict definitions, and standardized methods for diagnosing prognostic factors. Reporting on the locations, stages, and therapies of SSCs is strongly advised. Additionally, cohorts matched for HbA1c values and anti-diabetic medications may provide more accurate estimations of the impact of DM.

## Conclusion

Within the limitations of the study, diabetes is associated with significantly lower disease-specific and, with weaker evidence, lower overall survival rates in oral and oropharyngeal cancers, whereas laryngeal and nasopharyngeal subtypes are less affected. Diabetes is also associated with a twofold increased risk of osteoradionecrosis, and with weaker evidence for stroke development rate.

## Supplementary Information

Supplementary Material 1.

## Data Availability

The datasets used and/or analysed during the current study are available from the corresponding author on reasonable request.

## Declarations

All authors certify that they have participated sufficiently in the work to take public responsibility for the content, including participation in the concept, design, analysis, writing, or revision of the manuscript.
