# Supplementary material for: Diabetes is associated with elevated mortality, stroke, and osteoradionecrosis in squamous cell carcinomas of the head and neck: a systematic review and meta-analysis
Source: BMC Oral Health. 2026 Apr 22;26:1042. doi: 10.1186/s12903-026-08366-8 (PMC13270837; doi:10.1186/s12903-026-08366-8)
Supplement: Supplementary file 1 — Supplementary Material 1. [file 12903_2026_8366_MOESM1_ESM.docx]

**Supplementary figures**

**
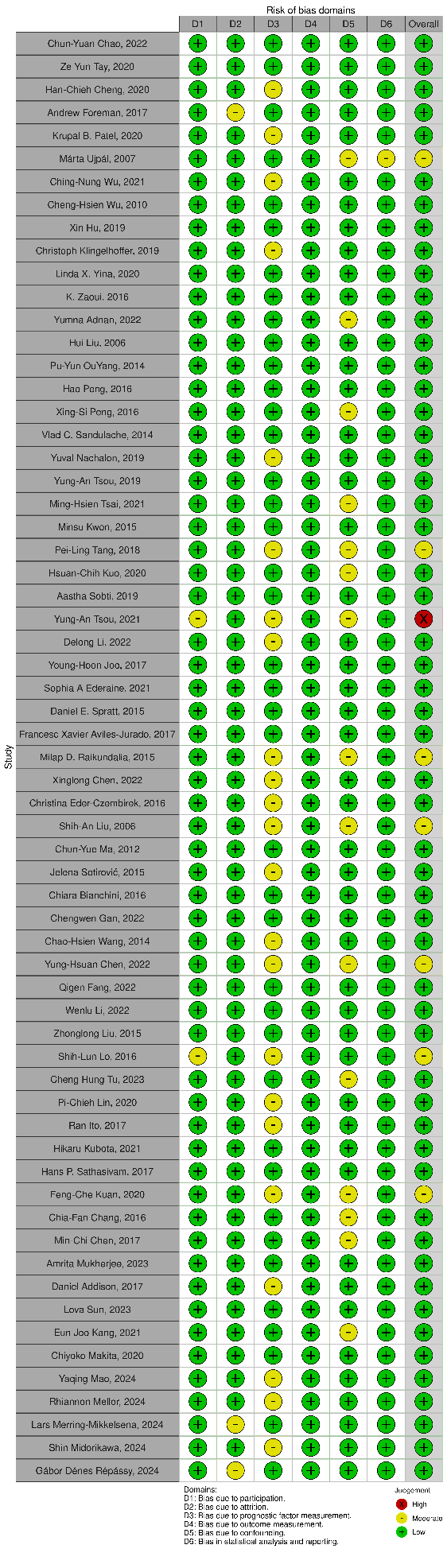
**

**Supplementary Figure 1.** Risk of bias assessment of the included studies based on the QUIPS tool.

**
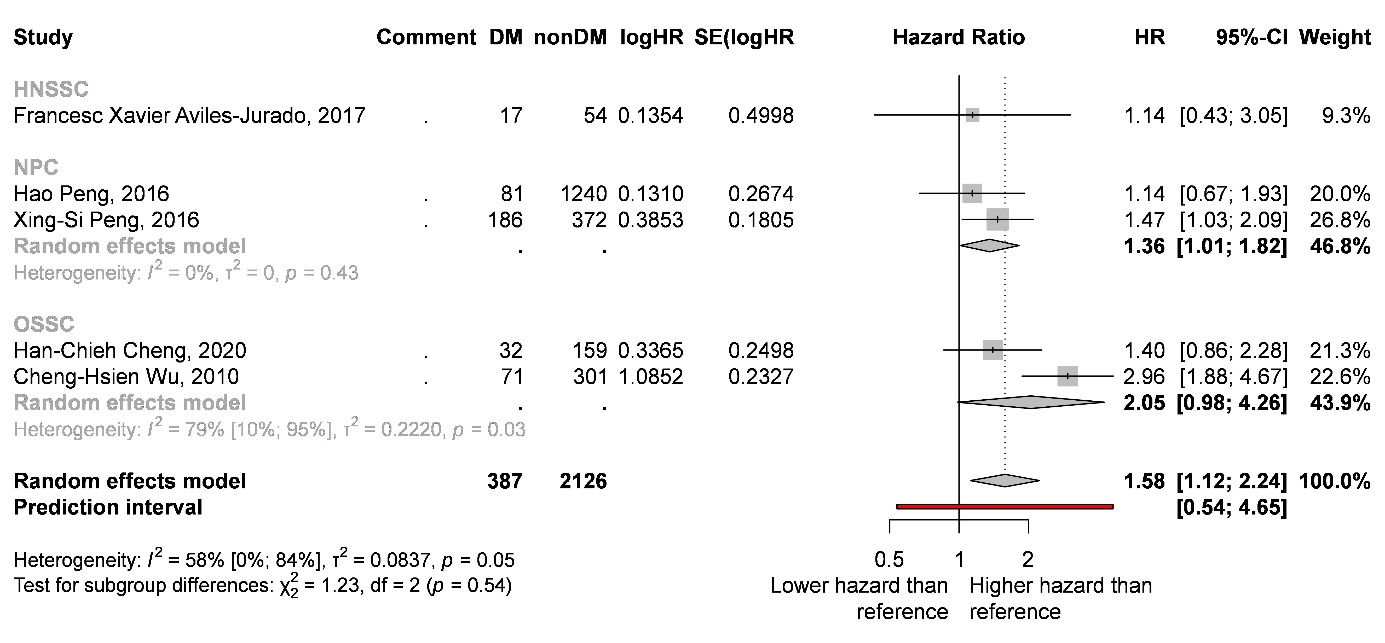
**

**Supplementary Figure 2.** Forest plot presenting disease-free survival in patients with diabetes compared to patients without diabetes, with subgroups based on different carcinoma locations.

**
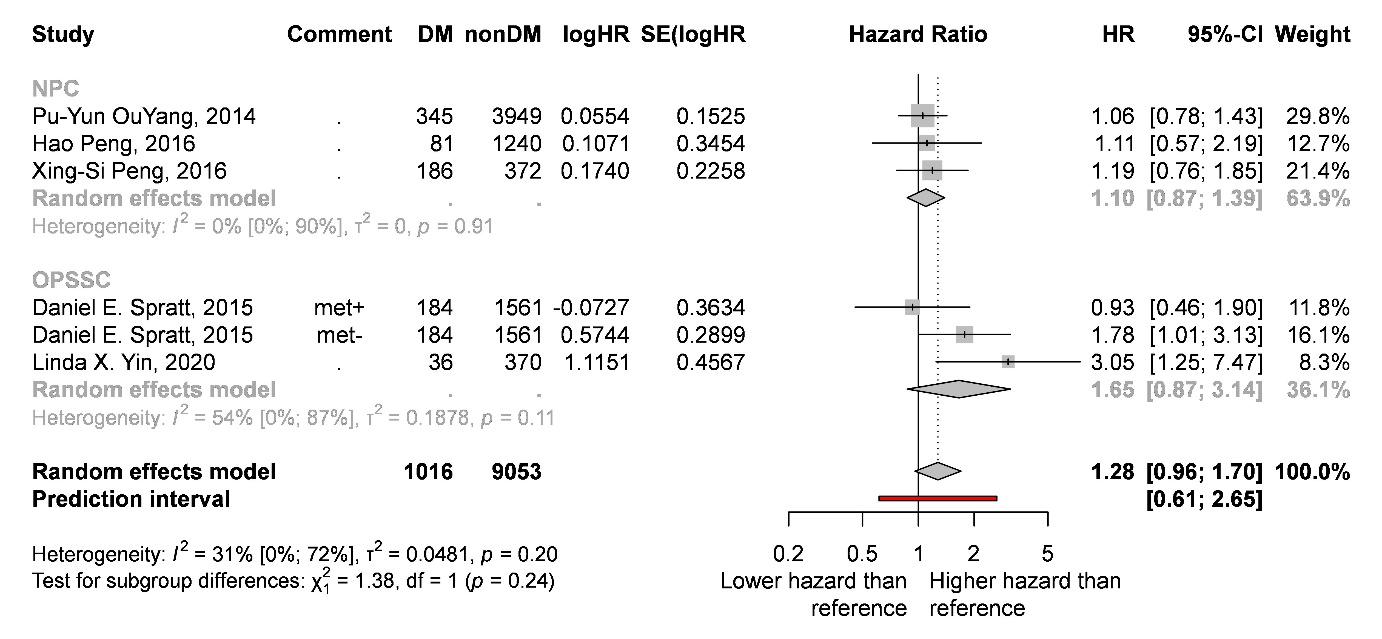
**

**Supplementary Figure 3.** Forest plot presenting distant metastasis free survival in patients with diabetes compared to patients without diabetes with subgroups based on different carcinoma locations.

**
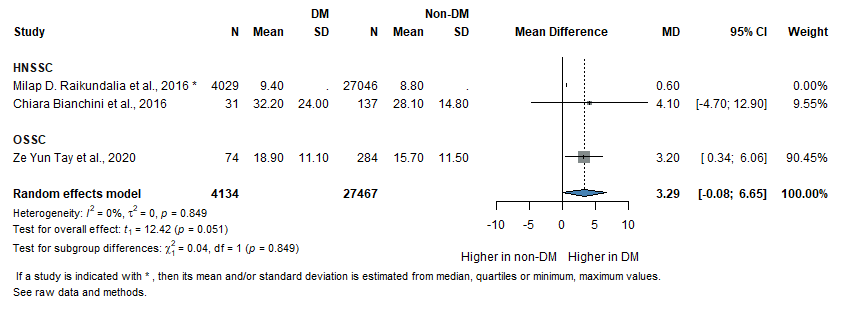
**

**Supplementary Figure 4.** Forest plot presenting the length of stay duration in patients with diabetes compared to patients without diabetes, with subgroups based on different carcinoma locations.


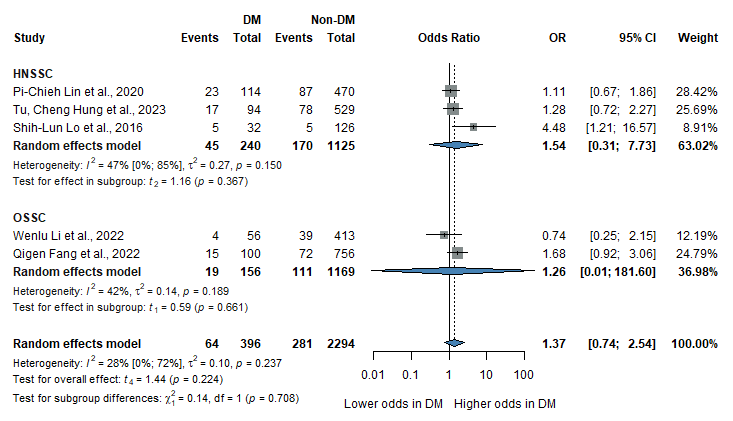


**Supplementary Figure 5.** Forest plot presenting flap complication rates in patients with diabetes compared to patients without diabetes, with subgroups based on different carcinoma locations.

**
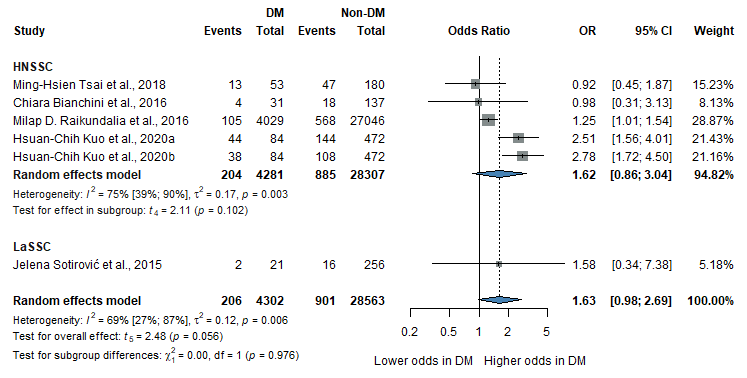
**

**Supplementary Figure 6.** Forest plot presenting wound infection rates in patients with diabetes compared to patients without diabetes, with subgroups based on different carcinoma locations.

**
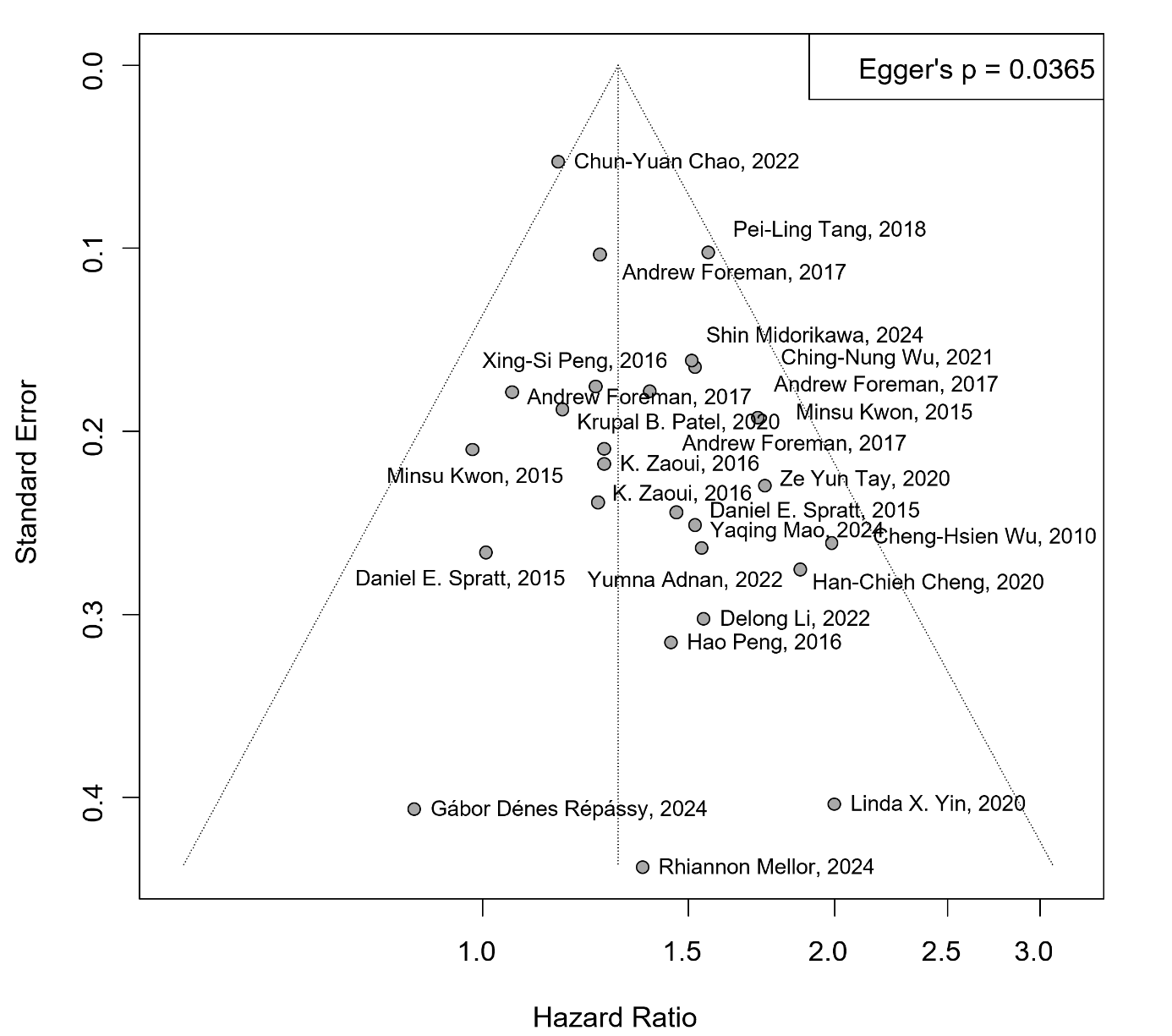
**

**Supplementary Figure 7.** Funnel plot presenting the possibility of publication bias in overall survival outcome.

**
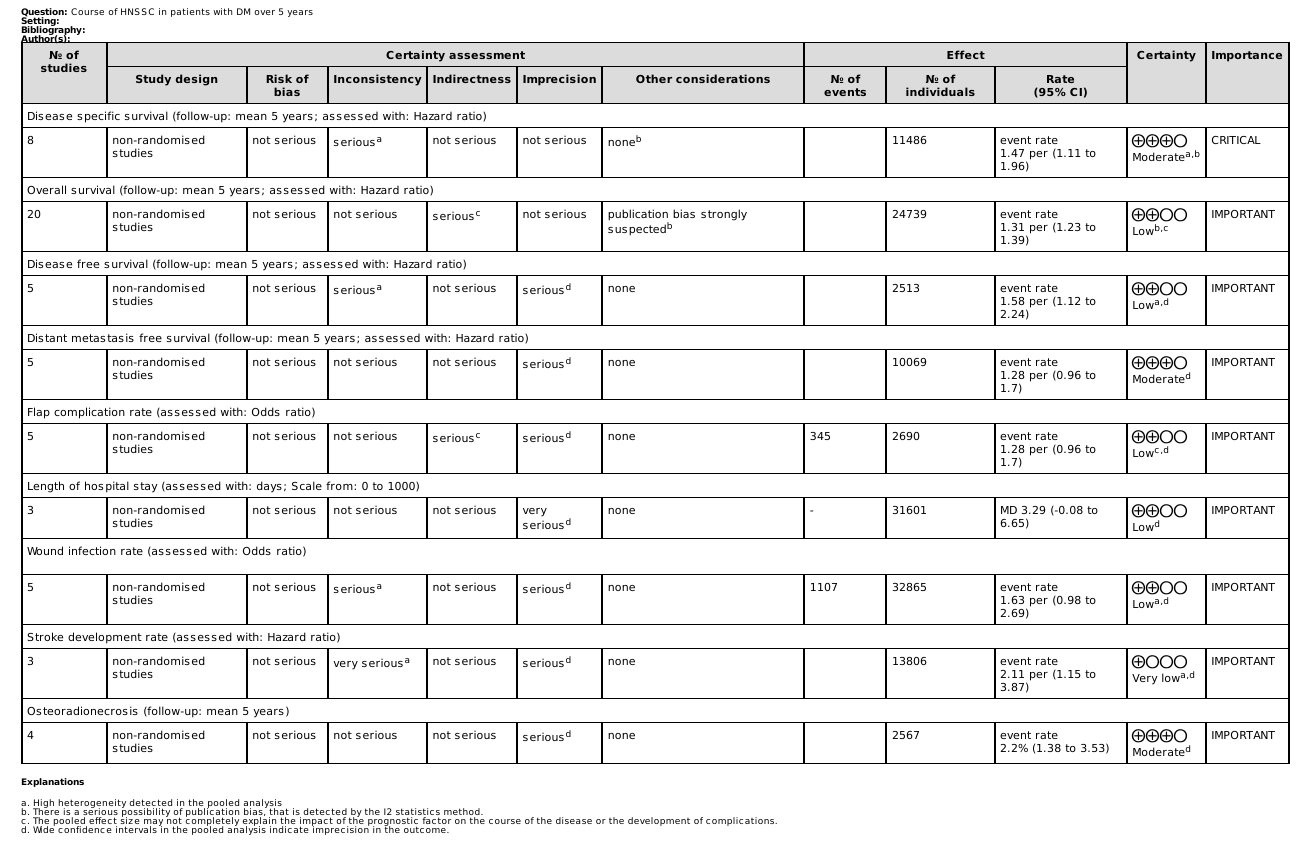
**

**Supplementary Figure 8.** Certainty of evidence assessment using the GRADEpro tool

**Supplementary tables**

| **Author (year)** | **Hazard ratio** | **Location** | **Adjustment** |
| --- | --- | --- | --- |
| Ze Yun Tay (2020) | 2.13 [1.299-3.5] | OSSC | Stage, differentiation, perineural invasion, extranodal extension, lymphovascular invasion, tumour depth, surgical margin involvement. |
| Ching-Nung Wu (2021) | 1.4 [0.92-2.14] | OSSC | Age and cancer stage. |
| Cheng-Hsien Wu (2010) | 2.16 [1.17-3.97] | OSSC | Age, sex, personal habits, fasting plasma glucose, tumour size, cervical nodal involvement |
| Pu-Yun OuYang (2014) | 0.98 [0.75-1.29] | NPSSC | NR |
| Minsu Kwon (2015) | 1.14 [0.68-1.92]* 0.79 [0.42-1,5]* | HNSSC | T3-4, N1-3, hypopharynx location, recurrence |
| Young-Hoon Joo (2018) | 2.11 [0.96-3.95] | HNSSC | Age, T and N stage, CVD in anamnesis |

**Supplementary Table 1** Multivariate analysis results on disease-specific survival.

| **Author (year)** | **Hazard ratio** | **Location** | **Adjustment** |
| --- | --- | --- | --- |
| Ze Yun Tay (2020) | 1.799 [1.12-2.85] | OSSC | Stage, differentiation, perineural invasion, extranodal extension, lymphovascular invasion, tumour depth, surgical margin involvement |
| Han-Chieh Cheng (2020) | 1.86 [0.93-3.71] | OSSC | NR |
| Ching-Nung Wu (2021) | 1.63 [1.17-2.25] | OSSC | Age, staging, treatment |
| Cheng-Hsien Wu (2010) | 2.22 [1.27-3.88] | OSSC | Age, gender, personal habit, fasting plasma glucose, tumour size, cervical nodal involvement |
| Christoph Klingelhoffer (2019) | 1.517 [0.874-2.634] | OSSC | Dental status, tumour stage, nodal stage, flap type, tracheotomy, neck dissection |
| K. Zaoui (2016) | 1.216 [0.677-2.185] | OSSC | Age, gender, tumour size, differentiation, lymph node involvement |
| Hao Peng (2016) | 1.495 [0.943-2.369] | NPSSC | Age, gender, pathology, T-stage, N-stage, pre-DNA, smoking, drinking, CHO, TG, LDL-C, HDL-C, hypertension, cardiovascular complications and chemotherapy |
| Xing-Si Peng (2016) | 1.23 [0.87-1.74] | NPSSC | T stage, N stage, overall stage and chemotherapy |
| Yuval Nachalon (2019) | 1.17 [NR] | LaSSC | Age, alcohol, disease stage |
| Minsu Kwon (2015) | 1.26 [0.82-1.93]; 0.95 [0.59-1.5] | HNSSC | Age, BMI, Smoking, T3-4, N1-3, Hypopharynx, Recurrence |
| Pei-Ling Tang (2018) | 1.6 [1.29-2.0] | HNSSC | Age group, geographic region, surgery,  radiotherapy, chemotherapy, AMI, COPD, number  of ED visits and number of admissions |
| Hsuan-Chih Kuo (2020) | 0.97 [0.68-1.4] | HNSSC | Stage, RT dose, infection |
| Aastha Sobti (2019) | 2.57 [1.09-6.07] | HNSSC | Age, male sex, alcohol, smoking, and ASA comorbidity score |
| Delong Li (2022) | 1.075 [0.938-2.984] | OSSC | NR |

**Supplementary Table 2** Multivariate analysis results on overall survival.

| **Author (year)** | **Hazard ratio** | **Location** | **Adjustment** |
| --- | --- | --- | --- |
| Cheng-Hsien Wu (2010) | 2.42 [1.49-3.92] | OSSC | Age, gender, personal habit, fasting plasma glucose, tumour size, cervical nodal involvement |
| Hao Peng (2016) | 1.0 [0.69-1.43] | NPC | Age, gender, pathology, T-stage, N-stage, pre-DNA, smoking, drinking, CHO, TG, LDL-C, HDL-C, hypertension, cardiovascular complications and chemotherapy |
| Xing-Si Peng (2016) | 1.44 [1.01-2.05] | NPC | T stage, N stage, overall stage and chemotherapy |
| Yuval Nachalon (2019) | 1.02 [NR] | LaSSC | Age, alcohol, disease stage |
| Francesc Xavier Aviles-Jurado (2017) | 1.85 [0.62-5.5] | HNSSC | ECOG index, Age |

**Supplementary Table 3** Multivariate analysis results on disease-free survival.

| **Author (year)** | **Hazard ratio** | **Location** | **Adjustment** |
| --- | --- | --- | --- |
| Linda X. Yin (2020) | 2.58 [1.06-6.26] | OPSSC | Overall pathologic stage |
| Pu-Yun OuYang (2014) | 1.1 [0.81-1.49] | NPSSC | Age, gender, smoking, drinking, hypertension, heart diseases, BMI, levels of total cholesterol, triglycerides, high density lipoprotein cholesterol and low-density lipoprotein cholesterol, titer of VCA-IgA and EA-IgA, histological type, T-stage, N-stage, chemotherapy and radiotherapy |
| Hao Peng (2016) | 1.195 [0.784-1.823] | NPSSC | Age, gender, pathology, T-stage, N-stage, pre-DNA, smoking, drinking, CHO, TG, LDL-C, HDL-C, hypertension, cardiovascular complications and chemotherapy |
| Xing-Si Peng (2016) | 1.18 [0.74-1.88] | NPSSC | T stage, N stage, overall stage and chemotherapy |

**Supplementary Table 4** Multivariate analysis results on distant metastasis free survival.

| **Author (year)** | **Odds ratio** | **Location** | **Adjustment** |
| --- | --- | --- | --- |
| Young-Hoon Joo (2018) | 4.09 [1.3-12.88] | HNSSC | Age, CVD, pulmonological, hepatic, renal diseases, CRT |
| Milap D. Raikundalia (2016) | 1.382 [NR] | HNSSC | Age, race |
| Xinglong Chen (2022) | 3.8 [2.65-5.47] | LaSSC | Age, sex, hypoalbuminemia, laryngectomy |
| Christina Eder-Czembirek (2016) | 2.2 [NR] | OSSC | BMI, alcohol, dentition |
| Shih-An Liu (2007) | 2.51 [1.409-4.475] | OSSC | Age, sex |
| Chun-Yue Ma (2012) | 6.076 [3.951-8.534] | OSSC | BMI, chronic obstructive  pulmonary disease, ASA score, CCI, and ACE-27 score |
| Chengwen Gan (2021) | 3.2 [2.22-4.18] | HNSSC | Location, dissection, flap failure, previous RT |
| Chao-Hsien Wang (2014) | 2.139 [1.05-4.337] | HNSSC | Age |

**Supplementary Table 5** Multivariate analysis results on surgical site infection.

| **Author (year)** | **Hazard ratio** | **Location** | **Adjustment** |
| --- | --- | --- | --- |
| Feng-Che Kuan (2020) | 1.46 [1.18-1.81] | OSSC | Hypertension, ischemic heart disease, atrial fibrillation, peripheral arterial occlusive disease, hyperlipidaemia and chronic kidney disease |
| Chia-Fan Chang (2016) | 1.37 [1.01-1.86] | OSSC | Sex, age, hypertension, previous stroke, arrhythmia, and coronary artery disease |
| Min-Chi Chen (2019) | 1.68 [1.21-2.34] | NPSSC | Age, treatment modality, comorbidities, and socioeconomic characteristics |
| Lova Sun (2023) | 1.15 [1.06-1.25] | HNSSC | Age, sex, race, and ECOG performance status, primary site, year of diagnosis, and T and N stage |
| Eun Joo Kang (2021) | 1.65 [1.33-2.05] | HNSSC | Sex, age, comorbidities |

**Supplementary Table 6** Multivariate analysis results on posttreatment stroke development.

| **Author (year)** | **Hazard ratio** | **Location** | **Adjustment** |
| --- | --- | --- | --- |
| Amrita Mukherjee (2023) | 2.25 [1.39-3.08] med+  1.94 [0.54-4.68] med- | HNSSC | Age, stage, hypertension, dyslipidaemia, treatment category |
| Lova Sun (2023) | 1.27 [1.16-1.38] | HNSSC | Age, sex, race, and ECOG performance status, primary site, year of diagnosis, and T and N stage |
| Eun Joo Kang (2021) | 1.15 [0.94-1.43] | HNSSC | Sex, age, comorbidities |

**Supplementary Table 7** Multivariate analysis results on posttreatment CVD complication.

| **Author (year)** | **Hazard ratio** | **Location** | **Adjustment** |
| --- | --- | --- | --- |
| Ran Ito (2017) | 4.08 [1.4-11.9] | OSSC | Age, sex, and BMI |
| Hans P. Sathasivam (2012) | 2.85 [1.54-5.28] | HNSSC | Tobacco usage, radiation dose, segmental mandibulectomy, marginal mandibulectomy, pre and postradiotherapy |

**Supplementary Table 8** Multivariate analysis results on posttreatment bone necrosis outcome.

| **Covariate category** | **Specific covariates included in multivariate models** |
| --- | --- |
| **Demographics** | Age, sex, race, geographic region |
| **Tumour characteristics** | T stage, N stage, overall stage, tumour size, tumour depth, differentiation, histological type, primary site, carcinoma location |
| **Lifestyle factors** | Smoking, alcohol consumption, personal habits, and tobacco use |
| **Comorbidities** | Cardiovascular disease, hypertension, COPD, chronic kidney disease, dyslipidaemia, diabetes-related comorbidities, renal/hepatic disease |
| **Metabolic factors** | BMI, cholesterol (CHO), triglycerides (TG), LDL-C, HDL-C, fasting plasma glucose, hypoalbuminemia |
| **Treatment-related variables** | Surgery, chemotherapy, radiotherapy, treatment modality, RT dose, neck dissection, tracheotomy, laryngectomy, flap type |
| **Cancer severity/performance status** | ECOG performance status, ASA score, CCI score, ACE-27 score |
| **Disease progression variables** | Recurrence, extranodal extension, lymphovascular invasion, perineural invasion, cervical nodal involvement |
| **Socioeconomic factors** | Socioeconomic characteristics, healthcare utilization (ED visits, admissions) |
| **Biomarkers/laboratory values** | Pre-treatment DNA titre, VCA-IgA, EA-IgA |
| **Dental/oral factors** | Dental status, dentition |
| **Other study-specific covariates** | Year of diagnosis, infection status, previous radiotherapy |

**Supplementary Table 9** Summary of covariates used in multivariate models across included studies.
